# Caspofungin and Polymyxin B Reduce the Cell Viability and Total Biomass of Mixed Biofilms of Carbapenem-Resistant *Pseudomonas aeruginosa* and *Candida* spp.

**DOI:** 10.3389/fmicb.2020.573263

**Published:** 2020-12-16

**Authors:** Luciana Fernandes, Bruna Nakanishi Fortes, Nilton Lincopan, Kelly Ishida

**Affiliations:** Department of Microbiology, Institute of Biomedical Sciences, University of São Paulo, São Paulo, Brazil

**Keywords:** antifungal, echinocandin, micafungin, polymicrobial biofilm, antibiofilm effect, resistance

## Abstract

*Pseudomonas aeruginosa* and *Candida* spp. are biofilm-forming pathogens commonly found colonizing medical devices, being mainly associated with pneumonia and bloodstream infections. The coinfection by these pathogens presents higher mortality rates when compared to those caused by a single microbial species. This study aimed to evaluate the antibiofilm activity of echinocandins and polymyxin B (PMB) against polymicrobial biofilms of carbapenem-resistant (CR) *Pseudomonas aeruginosa* and *Candida* spp. (*C. albicans*, *C. parapsilosis*, *C. tropicalis*, and *C. glabrata*). In addition, we tested the antimicrobial effect on their planktonic and monomicrobial biofilm counterparties. Interestingly, beyond inhibition of planktonic [minimum inhibitory concentration (MIC) = 0.5 μg/ml] and biofilm [minimum biofilm inhibitory concentration (MBIC)_50_ ≤ 2–8 μg/ml] growth of *P. aeruginosa*, PMB was also effective against planktonic cells of *C. tropicalis* (MIC = 2 μg/ml), and polymicrobial biofilms of CR *P. aeruginosa* with *C. tropicalis* (MBIC_50_ ≤ 2 μg/ml), *C. parapsilosis* (MBIC_50_ = 4–16 μg/ml), *C. glabrata* (MBIC_50_ = 8–16 μg/ml), or *C. albicans* (MBIC_50_ = 8–64 μg/ml). On the other hand, while micafungin (MFG) showed highest inhibitory activity against planktonic (MIC ≤ 0.008–0.5 μg/ml) and biofilm (MBIC_50_ ≤ 2–16 μg/ml) growth of *Candida* spp.; caspofungin (CAS) displays inhibitory activity against planktonic cells (MIC = 0.03–0.25 μg/ml) and monomicrobial biofilms (MBIC_50_ ≤ 2–64 μg/ml) of *Candida* spp., and notably on planktonic and monomicrobial biofilms of CR *P. aeruginosa* (MIC or MBIC_50_ ≥ 64 μg/ml). Particularly, for mixed biofilms, while CAS reduced significantly viable cell counts of CR *P. aeruginosa* and *Candida* spp. at ≥32 and ≥ 2 μg/ml, respectively; PMB was effective in reducing viable cells of CR *P. aeruginosa* at ≥2 μg/ml and *Candida* spp. at ≥8 μg/ml. Similar reduction of viable cells was observed for CAS (32–64 μg/ml) combined with PMB (2 μg/ml). These findings highlight the potential of PMB and CAS for the treatment of polymicrobial infections caused by *Candida* spp. and critical priority CR *P. aeruginosa*.

## Introduction

Biofilms are commonly defined as complex systems, comprising consortia of bacteria and/or fungi adhered to biotic or abiotic surfaces and lodged in a three-dimensional extracellular polymeric matrix (EPM) constituted mainly by secreted polysaccharides and proteins (Bandara et al., 2010; Deveau et al., 2018; Ibrahim et al., 2018). In this regard, most microorganisms are organized into biofilms, instead of living as planktonic cells, since biofilms offer protection against environmental, chemical, and mechanical stresses (Boudarel et al., 2018). Biofilm-derived-cells are phenotypically different from their planktonic counterparts; in fact, in the former, the reduction of microbial growth and metabolism can lead to the evasion of the immune system and increased resistance to antimicrobial agents (Ibrahim et al., 2018).

In hospital settings, microbial biofilms are relevant sources of infectious diseases, indeed, device-associated biofilm infections pose a considerable risk for patients (Desai et al., 2014; Ibrahim et al., 2018). In this regard, *Pseudomonas aeruginosa* and *Candida* spp. are among the most commonly found biofilm-forming pathogens and their biofilms are considered a risk factor for bacteremia/candidaemia, contributing to increased mortality rate (Ramage et al., 2005; Tumbarello et al., 2007; Vitkauskienė et al., 2010; Li et al., 2018; Lee et al., 2020).

Polymicrobial infections involving *P. aeruginosa* and *Candida* spp. have been described in wound infection, chronic lung disease, pneumonia associated with mechanical ventilation, and bloodstream infections (Klotz et al., 2007; Peleg et al., 2010; Trejo-Hernandez et al., 2014; Rodrigues et al., 2017).

Polymicrobial biofilms significantly alter treatment therapies and patient outcomes (Peters et al., 2012). For the treatment of *Candida* biofilms, only echinocandins and liposomal formulation of amphotericin B have been recommended (Pappas et al., 2016); however, the echinocandins are considered the first-line drug to treat candidemia related with biofilm formation. These antifungal agents exhibit non-competitive inhibition of the β-(1,3)-D-glucan synthetase enzyme, which catalyzes the polymerization of UDP-glucose to β-(1,3)-D-glucan, the main component of the fungal cell wall and biofilm EPM of *Candida* spp. (Denning, 2003). On the other hand, there are no specific recommendations for the treatment of *P. aeruginosa* biofilms. Polymyxins [polymyxin E (colistin) and polymyxin B (PMB)] are positively charged peptides used in the antimicrobial therapy, as a last option to treat infections caused by multidrug-resistant (MDR) Gram-negative bacteria, including carbapenem-resistant (CR) *P. aeruginosa*, which has become a serious health threat worldwide due to the limited options available for its treatment (Pogue et al., 2020).

The detection of β-(1,3)-D-glucan in the serum of patients suffering from *P. aeruginosa* infections has arisen an interest in evaluating the effect of echinocandins on this bacterial pathogen (Bazzi et al., 2013). In this respect, some studies have revealed the presence of β-(1,3)-D-glucan in *P. aeruginosa* as a periplasmic glucan polymer, and as an additional component of biofilm EPM (Lequette et al., 2007; Coulon et al., 2010; Beaudoin et al., 2012; Bazzi et al., 2013). Moreover, previous studies had demonstrated inhibitory effects of echinocandins on bacterial biofilms formed by *Staphylococcus aureus* and *P. aeruginosa* (Bazzi et al., 2013; Siala et al., 2016; Rasheed et al., 2018). Therefore, the *in vitro* activity of echinocandins and PMB against polymicrobial biofilms is worthy of investigation. In this study, we evaluated the activity of echinocandins and PMB, alone or in combination, against mixed biofilms of *Candida* spp. and CR *P. aeruginosa*.

## Materials and Methods

### Microorganisms

Clinical and environmental isolates of carbapenem-sensitive and CR *P. aeruginosa* (PAO1, ATCC 15442, ATCC 27853, 48-1997A, 141, 151, and 564-FC; Toleman et al., 2002; Fontes et al., 2011; Turano et al., 2016), and clinical isolates of *Candida* spp. (*C. albicans* SC5314, *C. paraposilosis* ATCC 22019, *C. tropicalis* ATCC 200956, and *C. glabrata* ATCC 2001) were used in this study. All isolates were maintained in brain heart infusion (BHI) broth with 20% glycerol at −80°C. *Candida* spp. and *P. aeruginosa* were recovered on Sabouraud dextrose and MacConkey agar, respectively, for 24 h at 37°C. Additionally, yeasts and bacteria were subcultured in Sabouraud dextrose or trypticase soy broths, respectively, for 24 h at 37°C, under agitation (150 rpm). All culture mediums were purchased from Becton, Dickinson and Company (New Jersey, United States).

### Antimicrobial Susceptibility Testing of Planktonic Cells

Antifungal drugs [i.e., caspofungin (CAS) and micafungin (MFG)] and PMB antibiotic were purchased from Sigma-Merck (Darmstadt, Germany), and stock solutions were prepared in dimethyl sulfoxide. Initially, the antimicrobial susceptibility profile of planktonic *P. aeruginosa* cells was determined by the disk diffusion method (Supplementary Table S1). Additionally, minimum inhibitory concentrations (MICs) of PMB and echinocandins were performed by broth microdilution methods, following CLSI guidelines for bacteria and fungi (Clinical and Laboratory Standards Institute, 2017, 2020). After broth microdilution testing, minimum microbicidal concentrations (MMCs) were determined as previously described (Freitas et al., 2018).

### Antimicrobial Susceptibility of Monomicrobial and Mixed Biofilms Formed by *Candida* spp. and Carbapenem-Resistant *Pseudomonas aeruginosa*

For the formation of polymicrobial biofilms, 50 μl of *Candida* spp. suspension at 1 × 10^7^ colony forming unit (CFU)/ml were incubated simultaneously with 50 μl of *P. aeruginosa* (PAO1, 151, and 48-1997A strains) suspension at 1 × 10^8^ CFU/ml, in RPMI 1640 medium buffered with 0.16 M MOPS (Sigma-Merck), using a 96-well flat sterile microplate. Monomicrobial biofilms were formed using 50 μl of each fungal or bacterial inoculum in wells containing 50 μl of medium. After 24 h of incubation at 37°C and under agitation (150 rpm), the monomicrobial or polymicrobial biofilms were washed with PBS twice and treated with several concentrations of CAS, MFG, or PMB (2–128 μg/ml) diluted in RPMI 1640 buffered with 0.16 M MOPS. Untreated biofilms and wells, containing only medium, were included in the assay, as microbial growth and sterility controls, respectively. In addition, the combination of CAS (32 or 64 μg/ml) with PMB (2 μg/ml) was tested against polymicrobial biofilms formed by *Candida* spp. and *P. aeruginosa* 151 strain. Microplates were incubated at 37°C, for 24 h at 150 rpm, the supernatant was removed, the biofilm washed in PBS twice, and the total biomass and cell viability were determined as described below. Assays were performed in quadruplicate in three independent experiments.

### Total Biomass Quantification

The total biomass (sessile cells plus EPM) of bacterial, fungal, and mixed biofilms was quantified using the violet crystal (VC) staining assay (Freitas et al., 2018). After the incubation period, biofilms were washed and fixed with 100 μl of methanol for 15 min, and then stained with 100 μl of 0.1% VC for 15 min. VC excess was removed and microplates were washed with distilled water and dried. The total biomass quantification was performed by adding 100 μl of 33% (v/v) glacial acetic acid as a CV solvent. Optical density (O.D.) at 590 nm of dissolved CV was measured in a microplate reader (Epoch 2 model, Biotek, Winooski, United States). The lowest concentrations of antimicrobials inhibiting 50% minimum biofilm inhibitory concentration (MBIC_50_) and 90% (MBIC_90_) of monomicrobial and polymicrobial biofilms were determined by calculating the inhibition percentage following the formula: 100 – (treated O.D. × 100/untreated O.D.), after subtraction of medium control O.D. Finally, MBIC_50_ and MBIC_90_ values were considered as a modal average from all results obtained.

### Viable Cell Count

The cell viability from polymicrobial biofilms after treatments with CAS or PMB (2, 8, 32, and 128 μg/ml) alone or in combination (32 or 64 μg/ml CAS plus 2 μg/ml PMB), compared with untreated biofilms, was evaluated by viable cell count assays. In brief, biofilms were washed and 100 μl of PBS was added to each well to remove the sessile cells by scraping and rinsing using a tip. The cells were transferred to a sterile microtube, sonicated at 42 KHz in an ultrasonic bath for 3 min, and serial dilutions (1:10) in PBS were performed and plated in a selective media (i.e., Cetrimide agar for *P. aeruginosa* and Sabouraud dextrose agar with 50 μg/ml chloramphenicol for *Candida* spp.) for CFU counting. Log CFU/ml values were calculated, and the final results were expressed as average and SD (Qu et al., 2016).

### Statistical Analysis

Statistical analyzes were performed using the GraphPad Prism v. 8 software (GraphPad, La Jolla, CA, United States), and values of *p* ≤ 0.05 were considered statistically significant.

## Results and Discussion

Antimicrobial susceptibility of planktonic bacterial mode of growth revealed that three *P. aeruginosa* strains (151, 141, and 48-1997A) exhibited a MDR profile (Magiorakos et al., 2012; Supplementary Table S1). In this regard, the MDR phenotype and carbapenem resistance was associated with SPM-1 metallo-*β*-lactamase production (Toleman et al., 2002; Fontes et al., 2011; Turano et al., 2016). Otherwise, *P. aeruginosa* strains ATCC 27853, ATCC 15442, and PAO1 were susceptible to all antibacterial tested, whereas 564-FC strain displays resistance to amikacin and ciprofloxacin (Supplementary Table S1). All *P. aeruginosa* strains were susceptible to PMB, whereas *Candida* spp. were susceptible to echinocandins (Table 1).

**Table 1:** Values of minimum inhibitory concentration (MIC) and minimum microbicidal concentration (MMC) of caspofungin (CAS), micafungin (MFG), and polymyxin B (PMB) against planktonic cells of *Candida* spp. and *Pseudomonas aeruginosa*.

Isolates	MIC or MMC (μg/ml)^a^					
	CAS^b^		MFG		PMB	
	MIC	MMC	MIC	MMC	MIC	MMC
*C. albicans* SC5314	0.03	0.25	≤0.008	0.12	> 8	>8
*C. tropicalis* ATCC 200956	0.03	1	≤0.008	0.25	2	>8
*C. parapsilosis* ATCC 22019	0.25	1	0.5	2	>8	>8
*C. glabrata* ATCC 2001	0.03	1	≤0.008	1	>8	>8
*P. aeruginosa* 141^c^	128	>128	>128	>128	0.5	0.5
*P. aeruginosa* 151^c^	128	>128	128	>128	0.5	0.5
*P. aeruginosa* 48-1997A^c^	64	>128	128	>128	0.5	0.5
*P. aeruginosa* 564-FC	>128	>128	>128	>128	0.5	0.5
*P. aeruginosa* ATCC 15442	64	>128	>128	>128	0.5	0.5
*P. aeruginosa* ATCC 27853	64	>128	>128	>128	0.5	0.5
*P. aeruginosa* PAO1 (ATCC15692)	64	>128	>128	>128	0.5	0.5

a MIC, the lowest concentration that inhibits 50% (for CAS and MFG) or 90% (for PMB) of microbial growth. MMC, the lowest concentration that kill >99.9% of microbial cells.

CAS, caspofungin; MFG, micafungin; and PMB, polymyxin B.

b No defined breakpoint values.

c Multidrug-resistant (MDR) strains (metallo-β-lactamase SPM-1-positive).

Polymyxin B inhibited only the planktonic growth of *C. tropicalis* ATCC 200956 at 2 μg/ml (Table 1). Interestingly, *C. tropicalis* ATCC 200956 isolate was previously described as resistant to AMB that has cross-resistance to azoles (Forastiero et al., 2013). On the other hand, among the echinocandins evaluated on planktonic bacterial cells, CAS showed bacteriostatic action on six *P. aeruginosa* strains, at 64–128 μg/ml, including CR MDR isolates 141, 151, and 48-1997A; whereas MFG showed inhibitory effects against CR *P. aeruginosa* 151 and 48-1997A strains, at 128 μg/ml (Table 1). Then, the selection of *P. aeruginosa* isolates was based on MDR profiles or susceptibility to clinically available antibiotics (MDR: 151 and 48-1997A, and susceptible: PAO1) for evaluation of antibiofilm effect of CAS and MFG against mono- and polymicrobial biofilms formed with *Candida* spp.

Monomicrobial biofilms of *P. aeruginosa* (PAO1, 151, and 48-1997A) were susceptible to PMB with MBIC_50_ ranging from ≤2 to 8 μg/ml (Table 2). However, only CAS, at 64 μg/ml, was able to inhibit *P. aeruginosa* 151 biofilm (Table 2). Previous studies have shown that MFG requires high concentrations (10 mg/ml) to have antibiofilm effect against *P. aeruginosa* isolates (Bazzi et al., 2013; Kissoyan et al., 2016) corroborating with our results which even higher concentration tested we did not observe any biofilm inhibition (Table 2).

**Table 2:** Minimum biofilm inhibitory concentrations (MBICs) of antimicrobials for monomicrobial and polymicrobial biofilms formed by *Candida* spp. and *Pseudomonas aeruginosa*.

Biofilms^a^	MBIC_50_/MBIC_90_(μg/ml)^b^		
	CAS	MFG	PMB
Monomicrobial
*Ca* SC5314	8/128	16/128	>128/>128
*Ct* ATCC 200956	4/128	≤2/32	32/>128
*Cp* ATCC 22019	64/128	16/64	>128/>128
*Cg* ATCC 2001	≤2/16	≤2/64	>128/>128
*Pa* PAO1	>128/>128	>128/>128	8/16
*Pa* 151	64/>128	>128/>128	≤2/16
*Pa* 48-1997A	>128/>128	>128/>128	≤2/16
Polymicrobial
*Ca* SC5314 + *Pa* PAO1	>128/>128	>128/>128	64/>128
*Ca* SC5314 + *Pa* 151	32/>128	32/>128	32/128
*Ca* SC5314 + *Pa* 48-1997A	64/>128	128/>128	8/>128
*Ct* ATCC 200956 + *Pa* PAO1	8/>128	16/>128	≤2/4
*Ct* ATCC 200956 + *Pa* 151	4/>128	8/>128	≤2/4
*Ct* ATCC 200956 + *Pa* 48-1997A	64/>128	32/>128	≤2/8
*Cp* ATCC 22019 + *Pa* PAO1	>128/>128	>128/>128	16/32
*Cp* ATCC 22019 + *Pa* 151	32/>128	64/>128	4/16
*Cp* ATCC 22019 + *Pa* 48-1997A	>128/>128	>128/>128	4/16
*Cg* ATCC 2001 + *Pa* PAO1	>128/>128	>128/>128	16/32
*Cg* ATCC 2001 + *Pa* 151	32/>128	32/>128	8/128
*Cg* ATCC 2001 + *Pa* 48-1997A	128/>128	64/>128	8/32

a *Ca*, *Candida albicans*; *Ct*, *Candida tropicalis*; *Cp*, *Candida parapsilosis*; *Cg*, *Candida glabrata*; and *Pa*, *P. aeruginosa.*

b MBIC_50_, the lowest concentration that inhibiting 50% of pre-formed biofilm; MBIC_90_, the lowest concentration that inhibiting 90% of pre-formed biofilm. CAS, caspofungin; MFG, micafungin; and PMB, polymyxin B.

As previously reported in the literature the echinocandins had a strong activity on *Candida* spp. biofilms but lesser than those observed on the planktonic cells corroborating with our findings (MBIC_50_ from ≤2 to 64 μg/ml vs. MIC ≤1 μg/ml; Tables 1, 2; Vila et al., 2016); whereas PMB was active only for *C. tropicalis* but at higher concentration (32 μg/ml; Table 2).

*Candida*-bacteria interactions are not a rare occurrence, and several studies suggest that *P. aeruginosa* and *C. albicans* interact with each other in the human body and in surfaces and frequently the coinfection leads to higher pathogenicity and mortality rates (Hogan and Kolter, 2002; Peleg et al., 2010; Trejo-Hernandez et al., 2014; Rodrigues et al., 2017). Although, *in vitro* studies have shown that *P. aeruginosa* forms a dense biofilm on *C. albicans* filaments and kills the fungus, *P. aeruginosa* neither binds to nor kills yeast-form *C. albicans*, even after prolonged incubation (Hogan and Kolter, 2002; Allison et al., 2016). In this study, we observed that *C. albicans* viability is totally preserved up to 2 days of interaction, and only on the 3rd day of fungus-bacterium interaction there was a reduction of fungal viability (Supplementary Figure S1). Therefore, antifungals and antibacterial treatments were performed on preformed 24-h mixed biofilms of *Candida* spp. and CR *P. aeruginosa*.

Our data showed that the polymicrobial biofilms formed by fungus-bacterium interaction were inhibited by antimicrobials CAS, MFG, and PMB at MBIC_50_ values ≥ MBIC_50_ values for monomicrobial biofilms (Table 2) corroborating with literature data that described the polymicrobial biofilms are more tolerant than their monomicrobial counterparties (Peleg et al., 2010). PMB was effective against polymicrobial biofilms of *P. aeruginosa* with *C. tropicalis* (MBIC_50_ ≤ 2 μg/ml), *C. parapsilosis* (MBIC_50_ = 4–16 μg/ml), *C. glabrata* (MBIC_50_ = 8–16 μg/ml), or *C. albicans* (MBIC_50_ = 8–64 μg/ml). Both echinocandins (CAS, 4 ≥ 128 μg/ml; MFG, 8 ≥ 128 μg/ml) reduced the total biomass of polymicrobial biofilms, especially for those formed by *Candida* spp. and *P. aeruginosa* strain 151, which were inhibited at the lowest MBIC values (Table 2). Therefore, additionally to the total biomass data, the cell viabilities of *Candida* spp. and *P. aeruginosa* (151 strain) from mixed biofilms were assessed after treatments with CAS or PMB.

There are several methods to evaluate the inhibition of biofilm formation besides VC staining (total biomass), such as XTT assay (metabolic activity of prokaryotic and eukaryotic cells) and CFU counting (cell viability), which are complementary and correlated (Mukherje et al., 2005; Wilson et al., 2017). Although XTT assay has been considered a gold standard method to evaluate the antibiofilm activity of compounds (Scudiero et al., 1988; Mukherje et al., 2005; Sabaeifard et al., 2014), in this study, we have used CFU counting to evaluate the viability of fungal and bacterial cells from single or mixed biofilms of *Candida* spp. and *P. aeruginosa*, since CFU counting allows to differentiate quantitatively (CFUs) fungus and bacterium viability inside of mixed biofilms by using selective culture media. In fact, CFU counting method has been often used in studies of inter-kingdom mixed biofilms (Qu et al., 2016; Santos et al., 2016; Kim et al., 2018).

**Figure 1:**
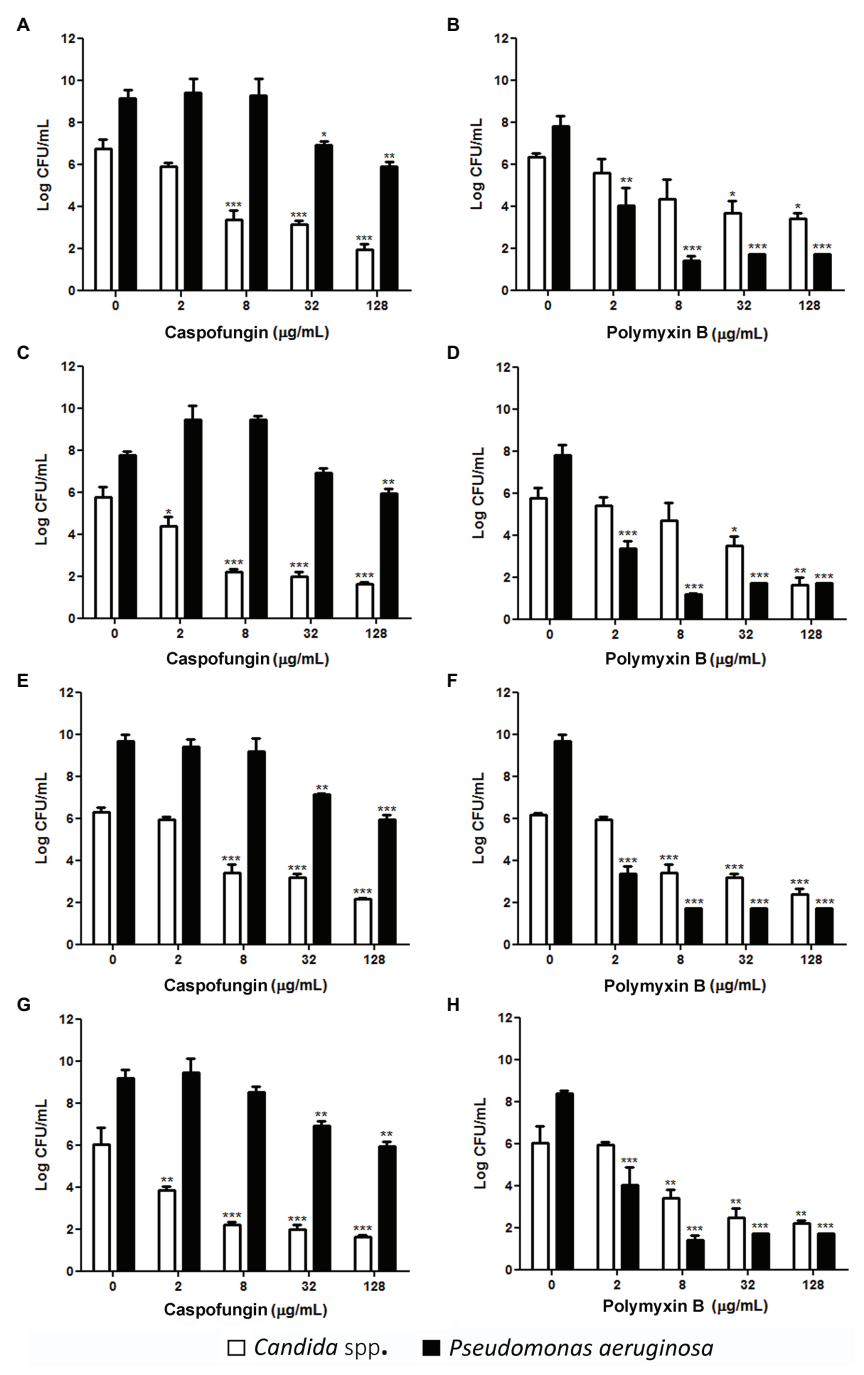
Count of viable cells of *Candida* spp. and *P. aeruginosa* 151 from polymicrobial biofilms after treatment with CAS or PMB. **(A)** and **(B)**: *C. albicans* SC5314; **(C)** and **(D)**: *C. tropicalis* ATCC 200956; **(E)** and **(F)**: *C. parapsilosis* ATCC 22019; and **(G)** and **(H)**: *C. glabrata* ATCC 2001. The results refer to the mean ± SD of three independent experiments in duplicate. ^*^ *p* < 0.05, ^**^ *p* < 0.01, and ^***^ *p* < 0.001 when compared with the respective untreated cells (group 0; on-way ANOVA with Dunnett’s posttest). Detection limit is 2 log colony forming unit (CFU)/ml.

Caspofungin, at concentrations ≥2 μg/ml, significantly reduced in up to 5 log, the number of viable counts of all *Candida* spp. in a dose-dependent manner, reaching undetectable levels at 128 μg/ml (*p* < 0.001, Figure 1), whereas CAS at concentrations ≥32 μg/ml decreased 2 log, the number of viable cells of *P. aeruginosa* 151 (*p* < 0.05, Figure 1). Notably, *P. aeruginosa* 151 – a CR and MDR strain – was inhibited by the echinocandins, both in planktonic growth mode or in monomicrobial and mixed biofilms (Tables 1, 2). The antibiotic PMB, at concentrations ≥2 μg/ml, reduced significantly the viability of *P. aeruginosa* 151, and at ≥8 μg/ml this reduction achieves undetectable levels in all mixed biofilms (*p* < 0.001, Figure 1). PMB at ≥8 μg/ml was also effective in reducing the viability of *C. glabrata* and *C. parapsilosis* in mixed biofilms with *P. aeruginosa* 151 (Figures 1F,H). Interestingly, mixed biofilms formed by hyphae/pseudohyphae-forming species (*C. albicans* and *C. tropicalis*) were susceptible only at concentrations ≥32 μg/ml PMB (Figures 1B,D).

Although echinocandins are known to be effective for the treatment of *Candida* biofilms, little is known about its effects on mixed biofilms formed by *Candida* spp. and bacterial pathogens. In fact, while antibacterial activity of echinocandins has been restricted to the evaluation of MFG against *P. aeruginosa* and *S. aureus* (Bazzi et al., 2013; Kissoyan et al., 2016; Lown et al., 2016; Rasheed et al., 2018), CAS has only been evaluated against *S. aureus*, so far (Siala et al., 2016; Moore et al., 2020). Interestingly, in this study, we report for the first time that CAS alone was able to inhibit and reduce the viability of *P. aeruginosa*, and this antibacterial activity was extended to critical priority MDR and carbapenemase-producing *P. aeruginosa* lineages belonging to the international sequence type ST277, which is endemic in Brazilian hospitals (Nascimento et al., 2016). In this regard, CAS inhibits 1,3-*β*-D-glucan synthesis in *Candida* spp.; therefore, since *P. aeruginosa* also has 1,3-β-D-glucan in its cell wall (Mennink-Kersten et al., 2008), the *in vitro* activity against planktonic and biofilm-forming *P. aeruginosa* could be expected.

The antifungal properties of polymyxins (PMB and colistin) on *C. albicans* and other fungal species have been previously described (Ogita et al., 2009; Zhai et al., 2010; Yousfi et al., 2019; Bidaud et al., 2020), but this activity has been directed against planktonic cells. In this study, while MIC values for planktonic *Candida* spp. were similar to those obtained in previous studies, we have observed additional inhibitory activity against *Candida* biofilms. The action of PMB on fungal cells is poorly understood, and some studies suggest effects on the fungal membrane (Zhai et al., 2010; Yousfi et al., 2019). This explanation could be plausible if we consider that polymyxins binds to the outer membrane of Gram-negative bacteria, where it complexes avidly with lipopolysaccharides showing an outer membrane-disorganizing action (Ayoub Moubareck, 2020). This primary damage in the outer membrane allows polymyxins to achieve the cytoplasmic membrane, where it causes leakage of cytoplasmic contents leading to bacterial death. Thus, it is evident that the membrane permeability changes immediately on contact with the drug. However, since activity of polymyxins is antagonized by Mg^2+^ and Ca^2+^ could be assumed that part of the action of this antibiotic is also due to the competitively displacing Mg^2+^ or Ca^2+^ from the negatively charged phosphate groups of membrane lipids (Ayoub Moubareck, 2020). Therefore, once cationic polymyxins interact with negatively charged moieties at the outer microbial cell structure, the negative charge of the fungal wall, conferred by mannoproteins that harbor phosphate groups (Ruiz-Herrera et al., 2006; Yousfi et al., 2019; Garcia-Rubio et al., 2020), would allow the coverage of fungal cells by polymyxins generating a positive charge on the cell surface with eventual disruption leading to antimicrobial effects. Interestingly, besides confer a negative charge to the cells, fungal glycoproteins containing abundant phosphodiester linkages provide hydrophilic or hydrophobic properties to the cell surface (Ruiz-Herrera et al., 2006; Yousfi et al., 2019), which could favor interaction with amphipathic polymyxin molecules. However, further studies should be conducted to clarify the exact mechanism of polymyxins against *Candida* spp.

The reduction of microbial viability of mixed biofilms after treatments with CAS or PMB is a relevant finding. The mean PMB maximum serum concentration (Cmax) at steady-state ranges from ~2 to 14 μg/ml after intravenous administration of clinically used dose (Avedissian et al., 2019), while CAS achieves 7–9 μg/ml after intravenous administration of recommended doses in adult patients (Groll et al., 2011). Then, concentrations ≥2 μg/ml of CAS or PMB were able to reduce significantly the viability of fungal and bacterial cells, respectively, from biofilms and achieving to undetectable levels at higher drug concentrations (Figure 1).

**Figure 2:**
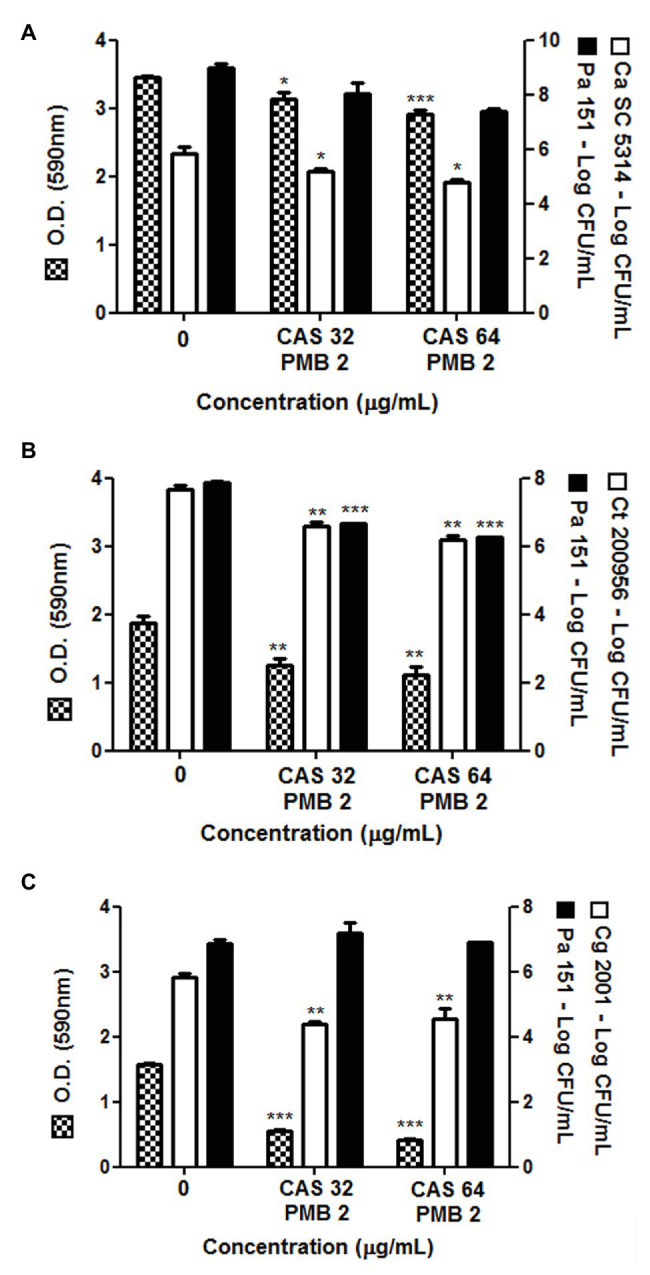
Total biomass and cell viability of pre-formed polymicrobial biofilms by *P. aeruginosa* 151 strain with different species of *Candida* treated with the combination of PMB (2 μg/ml) with CAS (32 or 64 μg/ml). Mixed biofilm of *P. aeruginosa* 151 formed with **(A)**: *C. albicans* SC5314, **(B)**: *C. tropicalis* ATCC 200956, and **(C)**: *C. glabrata* ATCC 2001. The results refer to the mean ± SD of three independent experiments in duplicate. ^*^ *p* < 0.05, ^**^ *p* < 0.01, and ^***^ *p* < 0.001 when compared with the respective untreated biofilms (group 0; on-way ANOVA with Dunnett’s posttest). Detection limit for count of viable cells is 2 log CFU/ml.

Here, we also evaluated the combination of CAS (32 or 64 μg/ml) with PMB (2 μg/ml) on mixed biofilms formed by *P. aeruginosa* strain 151 and *Candida* spp. (*C. albicans*, *C. tropicalis*, or *C. glabrata*; Figure 2). Both combinations were able to significantly reduce the total biomass of mixed biofilms, but mainly for those formed with *C. glabrata* ATCC 2001 (reduction of ca. 70%; Figure 2C). Moreover, a significant reduction of 1–2 logs in the viable fungal cells counting occurred in all fungus-bacterium interactions; however, for *P. aeruginosa* it occurred only when associated with *C. tropicalis* (Figure 2B). Although the antibiofilm effect of combinations of CAS with PMB was similar to the data obtained when monotherapies were used on mixed biofilms, the drug combinations are an important strategy to eradicate the bacterium-fungus biofilms, and then further studies should be conducted.

Synergistic effect of PMB combined with membrane-targeting antifungals (i.e., azoles and polyenes) or CAS, against only planktonic yeasts, has been showed (Moneib, 1995; Zhai et al., 2010; Adams et al., 2016; Rodrigues et al., 2017; Liu et al., 2019; Yousfi et al., 2019). Previous works described synergistic effect of PMB combined with azole agents and also combined with CAS against *Candida* spp., actuating as a facilitating agent for antifungals entry into the cell (Adams et al., 2016; Yousfi et al., 2019). In contrast, it has been suggested that echinocandin-mediated weakening of cell wall facilitates colistin targeting of membrane, which in turn enhance the antifungal activity of echinocandins (Zeidler et al., 2013).

Our work shows for the first time the combination of PMB with a conventional antifungal (CAS) against mixed biofilms formed by *P. aeruginosa* and *Candida* spp. CAS combined or not with PMB was able to disrupt the mono- and polymicrobial mature biofilms of *P. aeruginosa* and *Candida* spp. reducing the total biomass, as well as microbial viability. The combined use of echinocandins with another antibacterial agent could facilitate the penetration of this drug into deeper biofilm layers due to reduction of EPM produced by *Candida* cells. In addition, echinocandins are capable of non-competitively inhibiting the glycosyltransferase encoded by the *ndvB* gene, which acts in the production of the bacterial β-(1,3)-D-glucan (Beaudoin et al., 2012) presents in *P. aeruginosa* cell wall and EPM from biofilms contributing to mixed biofilm susceptibility (Bazzi et al., 2013). In this regard, combined treatment of MFG with ceftazidime, levofloxacin, ciprofloxacin, or aztreonam increases the survival rates of mice infected with *P. aeruginosa* (Kissoyan et al., 2016). In addition, CAS combined with fluoroquinolones acts as facilitator agent for penetration of the antibacterials into the *S. aureus* biofilms (Siala et al., 2016), as well as anidulafungin, another echinocandin agent, acts synergistically when combined with tigecycline against *in vivo* intra-abdominal mixed biofilms formed by *C. albicans* and *S. aureus* (Rogiers et al., 2018). Therefore, the activity of echinocandins on fungi and bacteria can be an important advantage in the treatment of polymicrobial infections in which *Candida* species are involved.

## Conclusion

Echinocandins, especially CAS, and PMB displayed *in vitro* activity against polymicrobial biofilms, reducing the total biomass and cell viability of *Candida* spp. and *P. aeruginosa*, including CR strains. Our results support the potential use of these antimicrobial agents to treat polymicrobial biofilm-mediated infections.

## Data Availability Statement

The original contributions presented in the study are included in the article/Supplementary Material, further inquiries can be directed to the corresponding author.

## Author Contributions

LF and BF performed the experiments, analyzed the results, and drafted the manuscript. KI and NL designed the experiments and wrote the manuscript. All authors contributed to the article and approved the submitted version.

### Conflict of Interest

The authors declare that the research was conducted in the absence of any commercial or financial relationships that could be construed as a potential conflict of interest.
