# Supplementary material for: Caspofungin and Polymyxin B Reduce the Cell Viability and Total Biomass of Mixed Biofilms of Carbapenem-Resistant Pseudomonas aeruginosa and Candida spp
Source: Front Microbiol. 2020 Dec 16;11:573263. doi: 10.3389/fmicb.2020.573263 (PMC7772422; doi:10.3389/fmicb.2020.573263)
Supplement: Supplementary file 1 [file Data_Sheet_1.pdf]

SUPPLEMENTARY MATERIAL

TABLE S1 Antimicrobial **s**usceptibility profile of *Pseudomonas aeruginosa* strains to antibacterials of clinical use.

P. aeruginosa strains	AMI	GEN	ATM	CPM	CAZ	CIP	IPM	MER	PTT
141	R	R	I	R	R	R	R	R	I
151	R	R	R	R	R	R	R	R	I
48-1997A	R	R	S	R	R	R	R	R	I
564-FC	R	S	S	S	S	R	S	S	S
ATCC 15442	S	S	S	S	S	S	S	S	S
ATCC 27853	S	S	S	S	S	S	S	S	S
PAO1	S	S	S	S	S	S	S	S	S

AMI, amikacin; ATM, aztreonam; CPM, cefepime; CAZ, ceftazidime; CIP, ciprofloxacin; GEN, gentamicin; IPM, imipenem; MER, meropenem; PTT, piperacillin-tazobactam. R, Resistant; I, intermediary; S, susceptible.

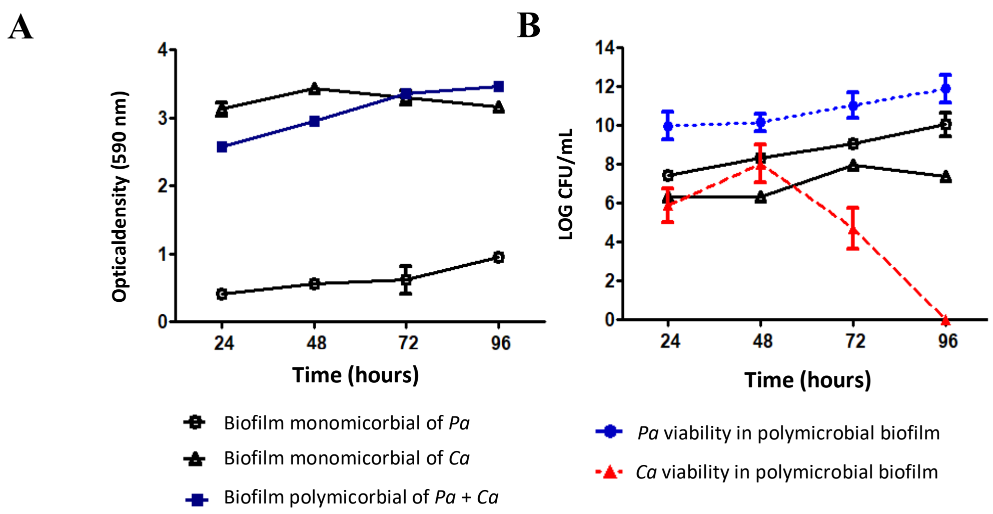

FIGURE S1 Formation kinetics of polymicrobial biofilms formed by *Candida albicans* SC5314 (*Ca*) and *Pseudomonas aeruginosa* PAO1 (*Pa*). **A:** total biomass of biofilms and **B:** cell viability of biofilms. The results indicate the mean ± standard deviation of three experiments in quadruplicate.
